# A High-Throughput *Candida albicans* Two-Hybrid System

**DOI:** 10.1128/mSphere.00391-18

**Published:** 2018-08-22

**Authors:** Floris Schoeters, Carol A. Munro, Christophe d’Enfert, Patrick Van Dijck

**Affiliations:** a VIB-KU Leuven Center for Microbiology, Leuven, Belgium; b KU Leuven Laboratory of Molecular Cell Biology, Institute of Botany and Microbiology, Leuven, Belgium; c Medical Research Council Centre for Medical Mycology at the University of Aberdeen, Institute of Medical Sciences, Aberdeen, United Kingdom; d Fungal Biology and Pathogenicity Unit, Department of Mycology, Institut Pasteur, INRA, Paris, France; Carnegie Mellon University

**Keywords:** Candida albicans, Candida albicans two-hybrid (C2H), ORFeome, high-throughput, protein-protein interactions, yeast two-hybrid

## Abstract

Candida albicans is a human fungal pathogen that does not follow the universal codon usage, as it translates the CUG codon into serine rather than leucine. This makes it difficult to study protein-protein interactions using the standard yeast two-hybrid (Y2H) system in the model organism Saccharomyces cerevisiae. Due to the lack of adapted tools, only a small number of protein-protein interactions (PPIs) have been detected or studied using C. albicans-optimized tools despite the importance of PPIs to understand cell biology. However, with the sequencing of the whole genome of C. albicans, the availability of an ORFeome collection containing 5,099 open reading frames (ORFs) in Gateway-adapted donor vectors, and the creation of a Gateway-compatible C. albicans-specific two-hybrid (C2H) system, it became possible to study protein-protein interactions on a larger scale using C. albicans itself as the model organism. Erroneous translations are hereby eliminated compared to using the S. cerevisiae Y2H system. Here, we describe the technical adaptations and the first application of the C2H system for a high-throughput screen, thus making it possible to screen thousands of PPIs at once in C. albicans itself. This first, small-scale high-throughput screen, using Pho85 as a bait protein against 1,646 random prey proteins, yielded one interacting partner (Pcl5). The interaction found with the high-throughput setup was further confirmed with a low-throughput C2H experiment and with a coimmunoprecipitation (co-IP) experiment.

**IMPORTANCE** Candida albicans is a major fungal pathogen, and due to the rise of fungal infections and emerging resistance to the limited antifungals available, it is important to develop novel and more specific antifungals. Protein-protein interactions (PPIs) can be applied as very specific drug targets. However, because of the aberrant codon usage of C. albicans, the traditional yeast two-hybrid system in Saccharomyces cerevisiae is difficult to use, and only a limited number of PPIs have been described in C. albicans. To overcome this, a C. albicans two-hybrid (C2H) system was developed in 2010. The current work describes, for the first time, the application of the C2H system in a high-throughput setup. We hereby show the usefulness of the C2H system to investigate and detect PPIs in C. albicans, making it possible to further elucidate protein networks in C. albicans, which has the potential to lead to the development of novel antifungals which specifically disrupt PPIs important for virulence.

## Abstract (precis)

Candida albicans is a major fungal pathogen, and due to the rise of fungal infections and emerging resistance to the limited antifungals available, it is important to develop novel and more specific antifungals. Protein-protein interactions (PPIs) can be applied as very specific drug targets. However, because of the aberrant codon usage of C. albicans, the traditional yeast two-hybrid system in Saccharomyces cerevisiae is difficult to use, and only a limited number of PPIs have been described in C. albicans. To overcome this, a C. albicans two-hybrid (C2H) system was developed in 2010. The current work describes, for the first time, the application of the C2H system in a high-throughput setup. We hereby show the usefulness of the C2H system to investigate and detect PPIs in C. albicans, making it possible to further elucidate protein networks in C. albicans, which has the potential to lead to the development of novel antifungals which specifically disrupt PPIs important for virulence.

## INTRODUCTION

Candida albicans is a pleiomorphic commensal diploid fungus and also an opportunistic pathogen leading to superficial infections but also life-threatening ones in immunocompromised patients (1, 2). C. albicans is among the four most deadly fungi, with mortality rates up to 50%, and responsible for the fourth most common nosocomial infection (1–3). In general, fungal infections are on the rise due to the advances in medical and intensive care, organ donation, implanted medical devices, and treatments of life-threatening (bacterial) infections (1, 4). Despite the rising impact of fungi, they are often ignored (1), and the development of antifungal drugs proves to be very challenging (5). At the moment, there is not a single antifungal vaccine on the market, only a limited number of antifungals are currently in use to combat fungal infections, and resistance is on the rise (1, 6).

In-depth knowledge about the function of proteins is crucial for the development of novel antifungals. A promising novel technique to develop very specific antifungals is targeting protein-protein interactions (PPIs) with small molecules (7–9). Information on PPIs is thus crucial for developing such antifungals, and the yeast two-hybrid (Y2H) system can play a pivotal role in this (10). For several organisms, large-scale or genome-wide Y2H studies to investigate PPIs have been published, contributing to an in-depth knowledge of, e.g., protein functions and cellular pathways (11–14). However, PPI studies are almost nonexistent for C. albicans (15, 16). The BioGRID database reports only 163 interactions (15), while the STRING database does not even mention C. albicans as a searchable organism (17), despite being one of the most studied fungal pathogens and even serving as a model organism (18). The lack of studied interactions is mainly due to C. albicans having a nonstandard CUG codon usage (translating CUG as serine rather than leucine most of the time), a low transformation efficiency, and unstable episomal plasmids, hindering the application of commonly used techniques such as the Y2H system (19–23). Besides the lack of PPI studies, the majority of genes/proteins are still uncharacterized (16, 19). Major contributing factors, next to the aberrant codon usage and lack of usable plasmids, are the C. albicans complex life cycle, having a heterozygous diploid nature, and the absence of a complete sexual cycle. These all make it hard to use standard genetic techniques (18, 20, 21, 24). In recent years, new technologies have been developed or adapted for use in C. albicans (18, 24, 25). Several of those techniques are focusing on PPIs (19, 26–28). One such technique is the specific C. albicans two-hybrid (C2H) system, which overcomes the problems associated with using the traditional Y2H system (19).

With the sequencing of the whole genome (16, 29–31), the availability of a Gateway-adapted C. albicans ORFeome containing 83% of the currently annotated coding sequences, and the adaption of the C2H system to a high-throughput setup, it became possible to screen PPIs on a larger scale (32). Here, we show the potential of the C2H system to detect PPIs by presenting the first large-scale application of this C2H system. Indeed, we have performed a high-throughput screen using a single bait protein (Pho85) against 1,646 random prey proteins in a mating approach setup. This led to the discovery of one new PPI that was further confirmed by a coimmunoprecipitation (co-IP) experiment. These results demonstrate the potential of the C2H system to screen for novel PPIs in a high-throughput setup, aiding the elucidation of protein networks in C. albicans. In-depth knowledge of PPIs and the function of proteins will aid the elucidation of many uncharacterized genes and gene products of C. albicans, leading to a better understanding of this commensal organism that can thrive as a major and deadly pathogen when the opportunity arises.

## RESULTS

### Generation of prey library.

With the available ORFeome collection (32), a preliminary, random library of prey-harboring C. albicans strains was generated. Using the LR reaction (Gateway system), 1,646 open reading frames (ORFs) were transferred from the pDONR207-XX (XX denoting a certain ORF) plasmids to the prey plasmid pC2HP-GC (32). Resulting prey plasmids pC2HP-XX were then transformed into mating-compatible strain SC2H3a-pWOR1. Transformants were selected on SC-Arg agar plates at 23°C for 5 days and stored in 96-well plates at −80°C. Each well contains one specific prey-harboring strain.

A bait plasmid containing the *PHO85* ORF was constructed and transformed into the mating-compatible strain SC2H3α-pWOR1. Transformants were selected on SC-Leu agar plates at 23°C for 5 days and stored in a 96-well plate, and so a matrix-approach C2H screen was possible.

### Setup of a high-throughput C. albicans C2H screen.

**FIG 1:**
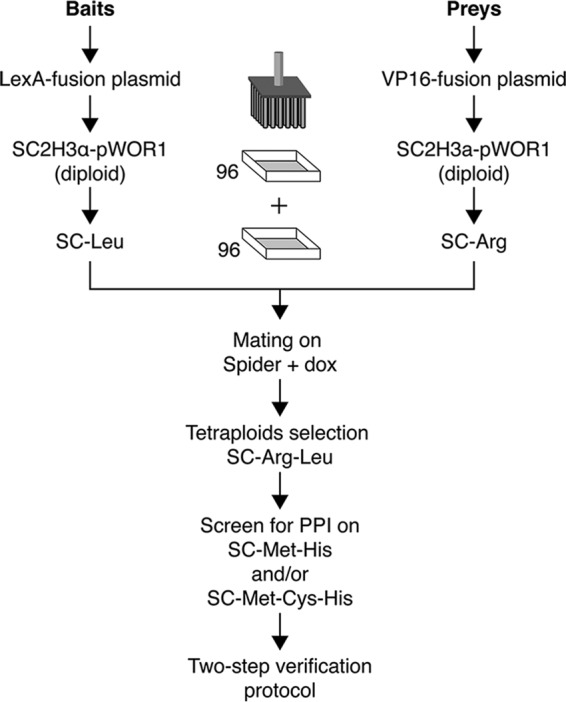
Schematic representation of the high-throughput C2H screen. Bait- and prey-harboring mating-competent C. albicans cells are spotted on SC-Leu or SC-Arg followed by replating on Spider-plus-Dox medium for mating. The mated cells (tetraploids) are then selected on SC-Leu-Arg prior to being screened for interaction. Interaction is screened on SC-Met-His or/and SC-Met-Cys-His medium. Growth on medium lacking histidine is indicative of interaction. Colonies growing faster and/or larger than background growth are kept for the next step, the two-step validation protocol. See the text for more details.

In 2010, the C2H system was described to study PPIs in C. albicans, leading to several novel interactions that were detected using this system. However, the system was limited to a low-scale setup (19). To overcome this limitation, the original C2H vectors were made Gateway compatible and inducible mating-competent C. albicans strains were created (32). The latter is important since the low transformation efficiency and unstable episomal plasmids make it difficult to perform a traditional pooled-style PPI screen. In the previously described study (32), the mating-compatible diploid strains harboring a bait or prey plasmid were mixed in liquid Spider medium without the addition of doxycycline (Dox) (see also below) (32). This approach is, however, less feasible for a high-throughput setup since mating in 96-well plates in liquid culture proved to be inefficient. In order to overcome this limitation, the mating of prey and bait was performed on Spider agar medium. Prey- and bait-harboring strains were manually transferred with a 96-replicate pinner, respectively, from SC-Arg and SC-Leu to Spider-plus-Dox agar plates and incubated for 5 to 6 days at 23°C to induce mating and the generation of tetraploids. The addition of doxycycline (Dox) is necessary for the induction of opaque switching by the *WOR1* regulator to stimulate mating by forming opaque cells (32). When doxycycline was omitted from the Spider medium, the mating efficiency dropped substantially even if Dox was added in the precultures of the prey and baits on SC-Arg or SC-Leu agar. Growing the cells at 23°C aids further in stabilizing the opaque cells, preventing them reverting to white cells that are less mating efficient (33). The cells were subcultured on SC-Arg-Leu agar plates to select for resulting tetraploids at 30°C for 2 to 3 days. Finally, the cells were transferred in duplicate to SC-Met-His and/or SC-Met-Cys-His medium and grown for up to 14 days at 30°C to screen for potential PPIs. Growth on medium lacking histidine is indicative of PPI. The selection between SC-Met-His and SC-Met-Cys-His medium depends on the background growth of the particular bait used. For baits showing a high background growth, it is advisable to use only SC-Met-His medium as this gives less expression of the bait (and prey) construct (34). A preliminary test in which the bait plasmid-harboring C. albicans strain is spotted on SC-Met-His and SC-Met-Cys-His should always be performed before starting with the C2H screen to detect autoactivation and strong background growth. It is important to keep in mind that the tetraploid strains formed during the high-throughput screen are less stable (and grow less well) than the SC2H3 diploid strain, cotransformed with bait and prey plasmids, during the two-step validation process (described below). It is thus possible that for the mating approach, SC-Met-Cys-His medium is needed, while for the cotransformation approach, SC-Met-His is required. To screen for potential interactions, plates were kept at 30°C for up to 14 days and (speed of) growth was scored relative to the background growth. All steps during the high-throughput screen were manually performed using a 96-replicate pinner since C. albicans forms dry, rubber-like colonies on Spider medium (35), making it harder to use an automated, robotic replicate system. Figure 1 provides an overview of the setup of a genome-wide two-hybrid screen.

**FIG 2:**
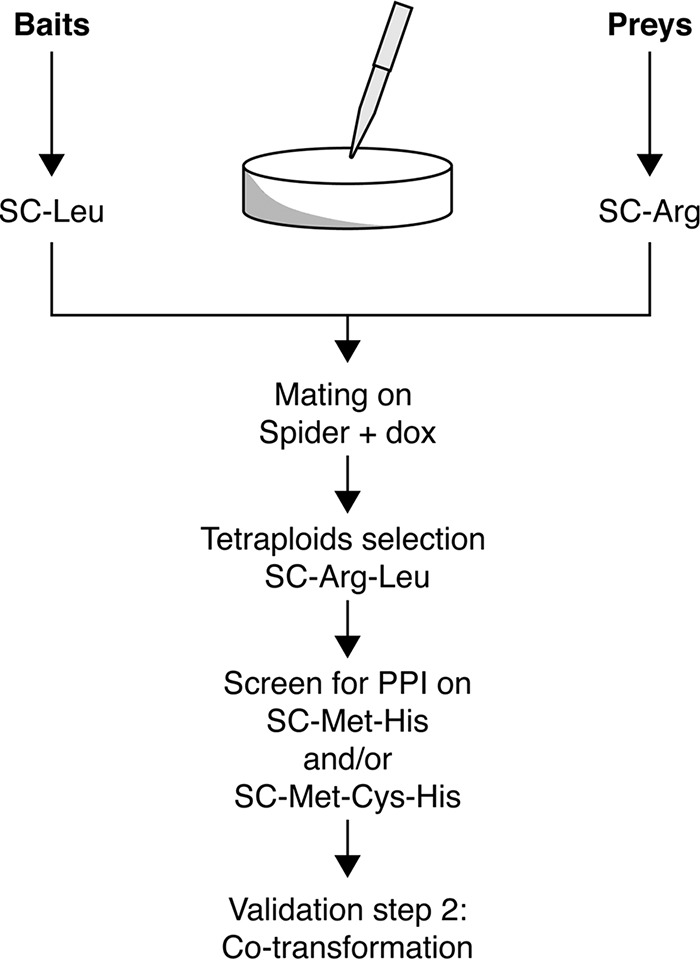
Schematic representation of the first step of the two-step validation protocol. Potential interacting bait and preys are manually spotted next to each other on Spider-plus-Dox medium followed by selection on SC-Leu-Arg. Successfully mated cells are then screened for potential interaction on SC-Met-His and SC-Met-Cys-His medium. Baits and preys that show potential interaction (growth on SC-Met-His and/or SC-Met-Cys-His) are then retested by a cotransformation in step 2 of the validation protocol. For this, both the bait and prey plasmids are cotransformed into a diploid SC2H3 strain and selected on SC-Leu-Arg, after which interaction is screened on SC-Met-Cys-His and/or SC-Met-His. In a final step, a spot assay can be performed.

False positives are a common problem with Y2H high-throughput screens. To decrease the amount of false positives, 3-aminotriazole (3-AT), an inhibitor of the *HIS3* gene product, is usually used (11); however, since the C2H system uses the *HIS1* marker (19) and not the *HIS3* marker, a stringent two-step validation protocol was designed. When growth was visible on SC-Met-His and SC-Met-Cys-His plates in the high-throughput C2H screen, indicating potential PPI, the corresponding bait- and prey-harboring strains were retested manually by spotting them next to each other, using a pipette tip, on fresh Spider-plus-Dox agar medium. After mating, the resulting tetraploids were grown on SC-Leu-Arg followed by selection on SC-Met-His and SC-Met-Cys-His to recheck the interaction (step 1). This extra step will significantly limit the number of false positives. In order to further validate the potential interactions identified and remove residual false positives, an independent C2H experiment was performed (step 2). In short, the bait and prey plasmids were cotransformed into the diploid SC2H3 strain and at least 3 independent transformants were further screened and checked for interaction followed by a final 5-fold dilution series spot assay on SC-Met-Cys-His and SC-Met-His (Fig. 2). The first step of the two-step validation protocol can be omitted when background growth during the initial high-throughput screen is almost absent. A potential third extra step to verify a potential interaction is to swap the bait and prey proteins. However, reversing bait and prey does not always lead to confirmation of the interaction and the results of such a swap experiment should be interpreted with care (11). The high-throughput screen followed by the two-step validation protocol already reduces false positives enormously (see below). However, to further confirm the identified interactions a different technique such as bimolecular fluorescence complementation (BiFC) (26) or co-IP (19) could be used. We have used a co-IP experiment as a final validation step.

### A high-throughput C2H screen using Pho85 as the bait protein.

To demonstrate the potential of C2H screening in a high-throughput setup, a random set of preys was screened against a specifically selected bait protein, Pho85. The selection of Pho85 as the bait was based on two criteria. The first important one is that Pho85 is an essential cyclin-dependent protein kinase (36). The second is the fact that the functional Saccharomyces cerevisiae Pho85 homologue has been shown to have several hundred interactors (37).

**FIG 3:**
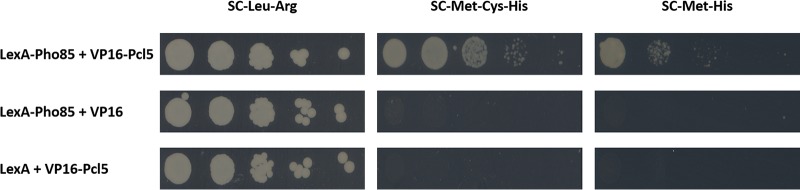
Spot assays using diploids created by cotransforming bait and prey plasmids in SC2H3, showing the interaction between bait Pho85 and prey Pcl5. No growth on selective medium (i.e., without histidine to select for interaction) is visible in strains expressing Pho85 and an empty prey plasmid (second row) or when only the Pcl5 prey is expressed (third row). Spot assays were performed in a 5-fold dilution. The MET3 promoter from which the hybrid genes are expressed is repressed in the presence of cysteine and methionine. Omitting both amino acids results in strong expression (middle column). Omitting only methionine results in a lower expression level, and as can be seen in the last column, there is less growth under this condition because of lower expression of bait and prey.

The high-throughput PPI screen was performed in a matrix setup in which bait protein Pho85 was mated with 1,646 random preys. Of these 1,646 prey-expressing strains, a total of 1,478 (90%) mated successfully with the Pho85 bait-expressing strain and formed tetraploids surviving on SC-Leu-Arg. From these 1,478 tetraploid strains, 15 showed promising growth on SC-Met-Cys-His and/or SC-Met-His medium. These 15 potential interactions were then manually retested. Only 5 of those 15 showed (weak) growth compared to the background growth and were kept for the second validation step (see Data Set S1 in the supplemental material). This final step led to one clear interaction partner: Pcl5, a cyclin (Fig. 3). Interestingly an interaction between the yeast orthologues of Pho85 and Pcl5 was already shown in S. cerevisiae (38, 39), and the Pcl5 protein has been shown to be a putative cyclin for Pho85 in C. albicans, similarly to its role in S. cerevisiae (40). Here, we provide evidence for the physical interaction between Pho85 and Pcl5 in C. albicans.

10.1128/mSphere.00391-18.1 DATA SET S1  Tab 1 gives an overview of the potential interactions found with the high-throughput screen followed by the two-step validation protocol leading to one validated interaction. Tab 2 gives an overview of all interactions that were shown by the yeast two-hybrid system with the *S. cerevisiae* Pho85 as bait. From the 23 interactors, there are five orthologues present in our *Candida* prey library, of which one (Pcl5) gives a strong interaction in *Candida*. A second protein (Pcl7) shows interaction with the cotransformation approach. Several orthologues of ScPho85 interactors are not yet present in our prey collection, and several other *S. cerevisiae* proteins do not have an orthologue (indicated with /). Tab 3 gives an overview of all *C. albicans* ORFs present in the current prey library. Download DATA SET S1, XLSX file, 0.1 MB. Copyright © 2018 Schoeters et al. 2018 Schoeters et al. This content is distributed under the terms of the Creative Commons Attribution 4.0 International license.

The four other potential interactions did not show growth on selective medium in the second step of the validation protocol. This, together with the reduction from 15 potential interactions to 5 in the first step of the two-step validation protocol, proves the strength and importance of this protocol. The two-step validation protocol clearly reduces false positives but can also lead to false negatives, which is another problem when using a Y2H system (11, 41, 42).

**FIG 4:**
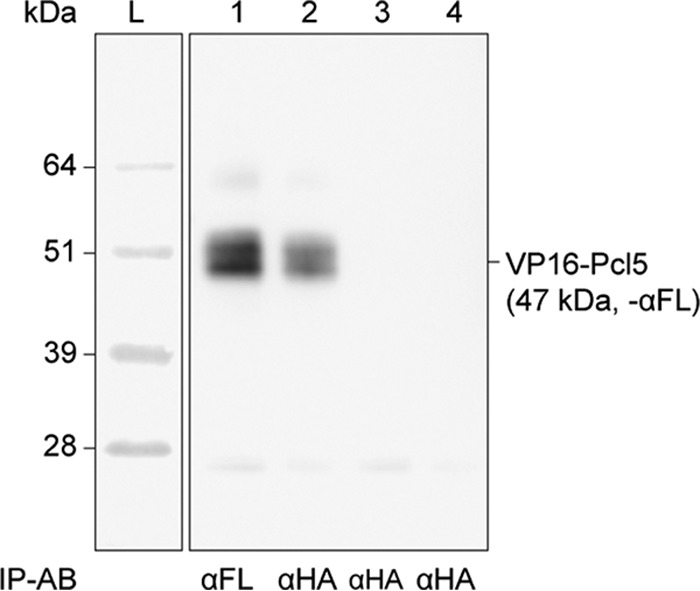
Coimmunoprecipitation of Pho85 and Pcl5. The interaction between bait Pho85 (LexA-HA-Pho85) and prey Pcl5 (VP16-FLAG-Pcl5) was confirmed in a co-IP experiment. Lane 1 shows the positive control of a co-IP of Pcl5 (IP with anti-FLAG). Lane 2 shows a co-IP of Pcl5 (IP with anti-HA). Lane 3 shows a control co-IP of strain SC2H3 transformed with the bait plasmid (pC2HB-Pho85) and an empty prey plasmid (pC2HP-GC) (IP with anti-HA). Lane 4 shows a control co-IP of strain SC2H3 transformed with an empty bait plasmid (pC2HB-GC) and the Pcl5 prey plasmid (pC2HP-Pcl5) (IP with anti-HA). The size ladder is shown in lane L. The VP16-FLAG-Pcl5 construct is approximately 47 kDa in size. The antibody (AB) used for Western blotting is indicated on the right side; the antibody for immunoprecipitation is indicated below the blot.

To further validate the identified interaction, a co-IP experiment was performed, confirming the interaction between Pho85 and Pcl5 (Fig. 4). In an independent experiment, we also switched bait and prey, creating Pcl5 bait- and Pho85 prey-containing plasmids, which were used to perform a cotransformation experiment followed by a spot assay. Growth was again detected, indicating interaction between the Pcl5 bait and the Pho85 prey, confirming the already-validated interaction between proteins Pho85 and Pcl5. Table 1 shows the identified interaction.

**TABLE 1:** Interaction between Pho85 bait and Pcl5 prey detected using high-throughput mating C2H screening and further checked with the two-step low-throughput confirmation protocol^a^

Bait protein (pC2HB-XX)			Prey protein (pC2HP-XX)			C2H result for spot assay of SC2H3 diploids	
Name	ORF no.	BCCM accession no.	Name	ORF no.	BCCM accession no.	SC-M-H	SC-M-C-H
Pho85	orf19.6846	LMBP 10482	Pcl5	orf19.4012	LMBP 10501	G (W)	G
Pcl5	orf19.4012	LMBP 10577	Pho85	orf19.6846	LMBP 10593	G (W)	G

^a^ During the high-throughput screen, the interaction was detected on an SC-Met-Cys-His agar plate. On the SC-Met-His agar plate, the growth was very weak and difficult to distinguish against the background growth. The interaction was then further validated with a low-throughput screen using tetraploids followed by a cotransformation and a spot assay of the diploid SC2H3 strain transformed with both the bait and prey plasmids. The interactions between bait Pho85 and prey Pcl5 were also shown using a co-IP experiment. We were also able to show the interaction with a cotransformation experiment when switching bait and prey. W, weak; G, growth; M, Met; C, Cys; H, His. See text for more details. BCCM accession numbers are given; plasmids are accessible at the Belgian Co-ordinated Collections of Microorganisms (BCCM; http://bccm.belspo.be/catalogues/genecorner-hosts).

## DISCUSSION

Despite C. albicans being an important human fungal pathogen and its whole genome being sequenced, the majority of C. albicans genes and proteins remain uncharacterized, largely limited by the aberrant codon usage (16). Yet, a better comprehension of the regulation and function of proteins can lead to a better understanding of C. albicans and its role in being both a commensal and pathogenic organism (43, 44). This can be achieved by an in-depth study of proteins and PPIs. Besides offering information on the biology of an organism, PPIs can also be exploited as the target of drugs such as antifungals (7, 9, 45, 46). Antifungals that can target the pathogen very specifically are increasingly needed with emerging fungal pathogens and rising resistance (1, 47). The creation of these very specific antifungals is a significant challenge given the host-like eukaryotic nature of fungal pathogens. Perhaps, the answer to this problem lies in the development of small molecules targeting PPIs (7, 47, 48). Several techniques are available to study PPIs, and since the first description of the Y2H system by Fields and Song (49), this system has been used to study thousands of PPIs for hundreds of organisms (50, 51). Originally, the Y2H technique was used in small-scale (low-throughput) studies in which specific interactions between often-known proteins were studied using S. cerevisiae as the host organism (52, 53). Since then, the system has been improved, changed, and adapted, leading to high-throughput screens and even genome-wide studies (12, 50, 51, 54). The Y2H system is, however, a limited system when studying C. albicans due to the differences in codon translation for the CUG codon. To overcome the difference in codon usage, the CUG codons are sometimes substituted when using the Y2H system (55); however, this is not ideal. To overcome this problem, a C2H system was created in 2010 (19). Here, we presented the first small-scale C2H high-throughput screening based on a mating approach. Using one specific bait, Pho85, we tested a total of 1,478 out of 1,646 potential (90%) interactions. The 168 potential interactions that could not be tested were caused by failed mating due to either the prey not growing on the original SC-Arg plate or the mating failing on Spider-plus-Dox plates. Current optimization in our lab has reduced this problem, and a mating efficiency up to 95% is achievable. To eliminate the problem of false positives, we applied a cautious two-step validation protocol. This reduced the potential interactions from 15 to 5 and finally yielded one interaction between the Pho85 and Pcl5 proteins. This interaction was further demonstrated in a low-scale (cotransformation) C2H setup and also by swapping bait and prey. Finally, it was validated by a co-IP experiment. The interaction was already described between the homologues in S. cerevisiae, and the C. albicans Pcl5 was further described in the work of Gildor et al. to be a putative Pho85 interactor (40). Our results now demonstrate the physical interaction in C. albicans between the cyclin-dependent kinase Pho85 and the cyclin Pcl5. Our lab is currently creating more prey- and bait-containing C. albicans mating-compatible strains, and ongoing C2H experiments in our lab are performed to screen several other baits against a random selection of +1,600 preys and are already showing promising novel interactions. Our prey collection contains five orthologues of S. cerevisiae proteins found to interact with *Sc*Pho85. Only for one of them could we show a clear strong interaction in C. albicans. Either this may reflect physiological differences between the two species, or the effect may also be caused by the technology itself. In S. cerevisiae, the Y2H system is performed with a strong promoter coupled with multicopy plasmids, resulting is very strong overexpression of the hybrid proteins. This is not the case in C. albicans, where each construct is integrated in the genome.

The two-step validation approach and the use of a manual 96-replicate pinner, however, currently limits the C2H system to smaller (manual) high-throughput screens. Although partly limited at the moment, it will be possible to scale up the C2H technique in the near future with adjustments so that an automated (robotic) approach can be used. Together with the completion of a genome-wide prey and bait collection, this will allow a genome-wide C2H screen of PPIs in C. albicans. Jointly with other techniques, such as the vesicle capture interaction assay (27), BiFC (26), tandem affinity purification (56, 57), and the “expanded genetic code” technique (28), the C2H system will bring us another step closer to elucidating the C. albicans protein interaction network and perhaps to the development of novel, very specific antifungals. The confirmed PPI found in this study will be uploaded to the BioGRID database (58), making it possible to extend the protein-protein interaction network in C. albicans. With the results presented here, we show the *Candida* community the potential of the C2H system to investigate PPIs. C2H high-throughput screening can be applied to detect multiple novel interactions, while the low-scale C2H system can be used to study putative interactions.

## MATERIALS AND METHODS

### Strains and growth conditions.

C. albicans strain SC2H3 (19) and its derivate mating-competent strains SC2H3a-pWOR1 and SC2H3α-pWOR1 (32) were used for the C2H system. These strains are deposited in the Belgian Co-ordinated Collections of Microorganisms (BCCM). Accession numbers are given in Table 2. Strain SC2H3 was used for the cotransformation validation step 2 of the high-throughput study. Strains SC2H3a-pWOR1 and SC2H3α-pWOR1 were used for the high-throughput study and validation step 1. Strains were cultured at 23°C or 30°C on YPD medium (1% yeast extract, 2% peptone, 2% dextrose, and 2% agar), Spider, or synthetic complete (SC) dropout medium from US Biological, respectively; SC-arginine (SC-Arg) for prey-plasmid-harboring strains; SC-leucine (SC-Leu) for bait-harboring strains; SC-Arg-Leu for strains with both prey and bait plasmids; SC-methionine-histidine (SC-Met-His); and/or SC-methionine-cysteine-histidine (SC-Met-Cys-His) to check for potential interactions. Preferably, high-quality medium is used (59). Omitting both methionine and cysteine will lead to a higher expression of the bait and prey protein (34). All synthetic complete medium was pH adjusted to 6.5 prior to autoclaving, and 2% agar was added. To induce the opaque state and mating, Spider medium was supplemented with doxycycline (Spider plus Dox) (50 µg Dox/ml) (32). Cells were grown at 23°C before and during mating; after mating, the cells were grown at 30°C for the high-throughput experiment. For the cotransformation experiments, cells were grown at 30°C.

**TABLE 2:** Yeast and bacterial strains used in this study

Strain	Genotype	Reference	BCCM accession no.
C. albicans			
SC2H3	SN152 *5xLexAOp-ADH1b/HIS1 5xLexAOp-ADH1b/lacZ*	19	LMBP 10468
SC2H3a-pWOR1	SC2H3-*MTLαD::FRT + pNIM1-WOR1-NAT1*	32	LMBP 10470
SC2H3α-pWOR1	SC2H3 + *MTLaD::FRT + pNIM1-WOR1-NAT1*	32	LMBP 10469
E. coli			
MC1061	*F^−^* Δ(*araA-leu*)*7697* [*araD139*]B/r Δ(*codB*-*lacI*)*3 galK16 galE15*(GalS) λ^−^ e14^−^ *mcrA0 relA1 rpsL150* *spoT1 mcrB1 hsdR2*	60	LMBP 472
DB3.1	*gyrA462 endA1* Δ(*sr1-recA*) *mcrB mrr hsdS20 glnV44* (=*supE44*) *ara14 galK2 lacY1 proA2 rpsL20 xyl5 leuB6 mtl1*	61	LMBP 4098

Escherichia coli strain MC1061 was used for plasmid propagation after the LR reaction took place. To propagate the plasmid pC2HB-GC or pC2HP-GC, *E. coli* strain DB3.1 was used. Strains were cultured at 37°C in LB supplemented with 50 µg/ml kanamycin (Kan) (prey or bait plasmid with ORF after LR reaction), 50 µg/ml kanamycin and 50 µg/ml chloramphenicol (Cm) (pC2HB-GC or pC2HP-GC propagation), or 50 µg/ml gentamicin (Gen) (pDONR207-XX). For solid medium, 2% agar was added. Table 2 gives more details. Competent E. coli cells were made using the CaCl_2_ method.

### Construction of the bait and prey two-hybrid plasmids and construction of the prey and bait mating-compatible library.

C. albicans ORFs were transferred from the pDONR207-XX plasmids into the bait and prey two-hybrid vectors pC2HB-GC and pC2HP-GC using the Gateway LR reaction, yielding pC2HB-XX or pC2HP-XX (32), respectively, according to the manual (Invitrogen) followed by transformation into chemically competent E. coli MC1061. Subsequently, the plasmids were digested with NotI prior to being transformed into the mating-compatible strain SC2H3a-pWOR1 (for preys) or SC2H3α-pWOR1 (baits) with a lithium acetate transformation (19, 32) followed by plating on SC-Arg (for preys) or SC-Leu (bait) medium. Resulting strains were stored at −80°C in 96-deep-well plates. When mating was not necessary, plasmids were cotransformed into strain SC2H3 (both prey and bait plasmids) with a lithium acetate transformation (19, 32).

### High-throughput two-hybrid analysis and validation of the potentially identified interactions.

The high-throughput screen was performed as described in detail in Results. The first steps were performed at 23°C to prevent the cells from switching from the opaque state to the white state. Doxycycline is added to the Spider medium to induce the opaque state. Further steps took place at 30°C. Plates have to be wrapped with Parafilm in all the steps except for the 2- to 3-day growth step at 30°C on SC-Leu-Arg medium. For the final step, to check for interaction, plates should be wrapped with at least two layers of Parafilm to prevent drying during a 14-day incubation at 30°C. The two-step validation protocol was used to greatly reduce the amount of false positives. An extra step in which bait and prey proteins are swapped can also be used as an extra control. The confirmation of an interaction found using the C2H can be achieved by using a different technique such as co-IP.

### Protein extraction for Western blotting and coimmunoprecipitation.

The protocol was based on reference 19 with minor adjustments. Briefly, overnight cultures were diluted to an optical density at 600 nm (OD_600_) of 0.5 in 50 ml and grown overnight to an OD_600_ of 5. Cells were collected by centrifugation at 4°C for 5 min at 3,500 rpm, and the pellet was washed twice with ice-cold phosphate-buffered saline (PBS; 140 mM NaCl, 2.7 mM KCl, 10 mM Na_2_HPO_4_, 1.8 mM KH_2_PO_4_ at pH 7.3). Supernatant was removed, and 500 μl lysis buffer (PBS; 2 mM EDTA, 10% glycerol, 0.1% Triton X-100, 50 mM Tris, 10 mM NaF, 4 mM Na_3_VO_4_, 10 mM β-glycerophosphate and one tablet of Complete protease inhibitor cocktail [Roche]) and glass beads were added. Cells were kept on ice for 5 min followed by bead beating (5 times for 30 s each at 6 m/s), being put on ice for 1 min between cycles, to extract proteins. A coimmunoprecipitation was performed with 500 µg protein and Pierce antihemagglutinin (anti-HA) magnetic beads (Thermo Fisher) by overnight incubation at 4°C. Following incubation, the beads were collected with a magnetic stand, washed three times with lysis buffer followed by adding SDS sample buffer (5×; 250 mM Tris-HCl, 10% SDS, 0.5% bromophenol blue, 1.4 M β-mercaptoethanol), and heated for 5 min at 95°C.

Proteins were separated by SDS-polyacrylamide gel electrophoresis on NuPAGE Novex Bis-Tris minigels (Invitrogen) followed by transfer to a nitrocellulose membrane (Hybond C extra; Amersham). Monoclonal anti-FLAG M2-peroxidase (horseradish peroxidase [HRP]) clone M2 was added for detection of coimmunoprecipitated proteins. Signals were detected using SuperSignal West Pico Luminol solution (Thermo Scientific). The immunoblots were imaged with Fujifilm LAS-4000 Mini and the accompanying software Image Reader LAS-4000 and Aida Image Analyzer v.4.22 (Life Sciences, Fuji, Photofilm Co., Ltd.).

As a positive control, a coimmunoprecipitation step was performed with anti-FLAG magnetic beads followed by detection of the protein using the anti-FLAG antibody.

### Data availability.

All plasmids and strains necessary to perform the C2H test are available from the BCCM collection (http://bccm.belspo.be). The collection/accession numbers are given in Tables 1 and 2. The pDONR207-XX plasmids containing the C. albicans ORFs (ORFeome) are available at the Centre International de Ressources Microbiennes (CIRM; https://www6.inra.fr/cirm_eng/) (32).

The “empty” Gateway-adapted bait and prey plasmids (pCHB-GC and pCHP-GC), the generated bait plasmids (pC2HB-Pho85 and pC2HB-Pcl5), and the interacting prey plasmids (pC2HP-Pcl5 and pC2HP-Pho85) (and their plasmid maps) are accessible from the Belgian Co-ordinated Collections of Microorganisms (BCCM; http://bccm.belspo.be). All LMBP accession numbers are provided in Tables 1 and 3.

**TABLE 3:** Plasmids used^a^

Plasmid	C. albicans selection marker	E. coli selection marker(s)	Parental plasmid	Reference	BCCM accession no.
pC2HB-GC	CmLEU2	Kan, Cm	pC2HB	32	LMBP 9242
pC2HP-GC	CdARG4	Kan, Cm	pC2HP	32	LMBP 9244
pDONR207-XX		Gen	pDONR207	32	

^a^ XX denotes a specific ORF inserted in the Gateway donor vector pDONR207 from the C. albicans ORFeome project (32). For the creation of the C2H prey and bait library, ORFs were transferred from the pDONR207 plasmid to the pC2HB-GC or pC2HP-GC vectors using the Gateway LR reaction system according to the protocol in the Invitrogen manual.
